# A Spatially Resolved Mechanistic Growth Law for Cancer Drug Development Predicting Tumor Growing Fractions

**DOI:** 10.1158/2767-9764.CRC-22-0032

**Published:** 2022-08-02

**Authors:** Adam Nasim, James Yates, Gianne Derks, Carina Dunlop

**Affiliations:** 1 Department of Mathematics, University of Surrey, Guildford, United Kingdom.; 2 Oncology R&D, AstraZeneca, Cambridge, United Kingdom.

## Abstract

Mathematical models used in preclinical drug discovery tend to be empirical growth laws. Such models are well suited to fitting the data available, mostly longitudinal studies of tumor volume; however, they typically have little connection with the underlying physiologic processes. This lack of a mechanistic underpinning restricts their flexibility and potentially inhibits their translation across studies including from animal to human. Here we present a mathematical model describing tumor growth for the evaluation of single-agent cytotoxic compounds that is based on mechanistic principles. The model can predict spatial distributions of cell subpopulations and account for spatial drug distribution effects within tumors. Importantly, we demonstrate that the model can be reduced to a growth law similar in form to the ones currently implemented in pharmaceutical drug development for preclinical trials so that it can integrated into the current workflow. We validate this approach for both cell-derived xenograft and patient-derived xenograft (PDX) data. This shows that our theoretical model fits as well as the best performing and most widely used models. However, in addition, the model is also able to accurately predict the observed growing fraction of tumours. Our work opens up current preclinical modeling studies to also incorporating spatially resolved and multimodal data without significant added complexity and creates the opportunity to improve translation and tumor response predictions.

**Significance:**

This theoretical model has the same mathematical structure as that currently used for drug development. However, its mechanistic basis enables prediction of growing fraction and spatial variations in drug distribution.

## Introduction

Preclinical evaluation of drug efficacy plays a fundamental role in the development of oncological treatments, with the aim being to predict pharmacologically active drug concentrations and guide dose exploration in the clinic. Data for these studies come from longitudinal measurements of tumor volume in animal models with specific targets investigated by the use of both cell-derived xenograft (CDX) and patient-derived xenografts (PDX); ref. 1. Central to these preclinical studies are *mathematical* models used to describe the tumor dynamics, fit the experimental data, and evaluate the antitumor effect. These models usually take the form of simple growth laws for tumor volume. There are many such models which can satisfactorily capture the dynamics (2–7) so that for any given dataset it is in practice very difficult to distinguish between them (4–6, 8–10). This is compounded by the fact that typically these growth functions are purely empirical descriptions of the data, not founded in a mechanistic description of the physiologic process.

The extension of growth law modeling in preclinical trials into mechanistically based modeling would have significant advantages. It could aid discrimination between models as models could be chosen by mechanism rather than only by fit. It would also enable key physiologic processes to be better integrated in trial design and encourage the use of a broader range of data beyond tumor volume. Indeed, current preclinical growth laws typically do not even account for the variations in growth fraction dynamics and hypoxia that have been shown to be key to determining treatment response (11–14). Mathematically, there have been significant advances in mechanistic models of tumor physiology, with simulations now possible even at the level of entire vasculatures (15). However, from a practical perspective, these more complex models raise significant additional challenges, which inhibits their incorporation into current preclinical workflows. They typically are based on a detailed knowledge of, for example, tumor geometry, which is not typically acquired in preclinical trials, and require significant computational simulations. In addition, they will have more parameters, which makes it challenging to parameterize them. This raises concerns of parameter identifiability, although this may not necessarily inhibit their predictive application (16).

Here, we present a mechanistic and spatial mathematical model for tumor growth based on a well-accepted mathematical framework coupling spatial diffusive processes to tumor growth (17). Significantly, its spatial underpinning means that it predicts the size of the growing fraction of the tumor over time. Under the assumption that the tumor is spherical we show that this model can be expressed as a growth law of a similar type to that currently used in the pharmaceutical industry. It thus can be fitted and analyzed using current industry-standard methods. We demonstrate the ease of use by comparison with a current commonly adopted growth law and validate both the models using standard methods on CDX and PDX data. Another advantage of the model is it can predict tumor growth fraction, which we here validate by endpoint histology. The new framework offers further significant advantages such as being able to account for spatial gradients in drug distribution. Together, these demonstrate that mechanistic models can be made fit-for-purpose to be incorporated into the current preclinical trial workflow and can enable the incorporation of data with spatial resolution as this is more routinely obtained.

## Materials and Methods

### Mathematical Models for Tumor Growth and Treatment Effect

Mathematical models as used in preclinical studies typically can be expressed as a rate equation for the tumor volume 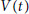 of the form



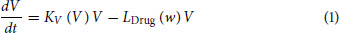



with the initial tumor volume 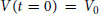, a fit parameter. The function 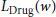 describes cell loss as a function of the drug concentration 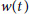, most often 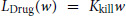 which represents exponential loss of tumor volume. The function 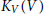 represents the net growth rate, with a range of different forms used in preclinical studies (refs. 3, 6, 18; Table 1) with the form often optimized for data fitting. For example, where growth is observed to be linear 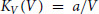, whereas exponential growth is captured by 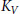 constant. Although all the models from Table 1 have successfully been used to fit preclinical data, it is often challenging to distinguish them in terms of quality of fitting (5, 6, 8–10). However, there are some key structural differences. The first five (linear, exponential, logistic, Gompertz, and exponential linear) are commonly used empirical growth laws. The surface growth model (19) and the proliferative rim model (20), are less widely adopted, and while mechanistic in foundation, each assume proliferation is restricted to a fixed unchanging region of the tumor. As such these models can only be expected to mechanistically approximate the tumor dynamics in specific circumstances and for limited times. A significant drawback of all the growth models in Table 1, except the Gompertz and logistic models, is that they predict unbounded growth as time increases, which is not experimentally observed. However, the listed models may still adequately describe animal-derived data, as the data cannot be obtained over long enough timescales for any growth retardation to be observed. However, in clinical studies where the observation timescales are longer, it is less clear that this would hold.

**TABLE 1:** Example growth models used in preclinical modeling

Model	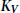	Parameters^a^
Linear	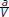	2
Exponential	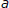	2
Logistic	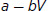	3
Gompertz	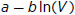	3
Exponential-linear (2)^b^	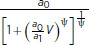	4^c^
Surface growth (19)	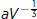	2
Proliferative Rim (20)	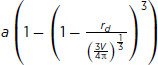	3

^a^The number of fit parameters for each model includes the initial tumor volume.

^b^In the exponential-linear model, 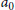 and 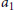 are the growth rates in the exponential and linear phases, respectively with 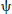 describing the transition between phases.

^c^ 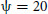 is typically taken, ensuring a fast transition, so that it is effectively a three parameter system.

**FIGURE 1:**
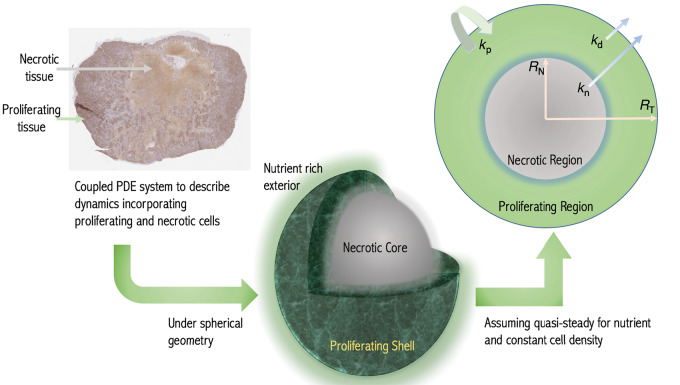
Schematic of the modeling concept. A growing tumor is modeled as a proliferating shell encapsulating a necrotic (nonproliferative core) with the boundaries between regions determined dynamically by considering nutrient diffusion. The assumed geometry and model variables and parameters are labeled in the cross-section with 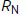 and 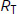 being the necrotic and total tumor radii, respectively. (Histologic image—a day-24 CDX xenograft with Ki-67 stain.)

An alternative approach to modeling tumor growth outside of pharmaceutical drug development has been to model the spatial diffusion of nutrients within tumors directly. It is assumed that nutrient availability determines the status of the cell, with cells experiencing low nutrient levels undergoing necrosis, a form of cell death. This results in a map of the tumor with different sized cellular compartments for, for example, proliferative and nonproliferative necrotic (dying) compartments determined responsively from the environmental conditions (17, 21), see Fig. 1, as is observed experimentally (22). The size of the compartments thus alters dynamically and is determined organically from the tumor growth, generating predictions for the size of each compartment over time. This basic framework has been extensively built upon in the mathematical literature (23, 24). The increasingly complex models are typically expressed in terms of partial differential equations which are either solved numerically themselves or coupled into finer scaled simulations.

We adopt this diffusion-based framework and make simplifying assumptions appropriate to the preclinical context. We thus reduce the model to a growth law of the form Eq. (1). The method is based on assuming a spherical tumor with the nutrient penetration within the tumor determined from the diffusion equation. From the diffusion equation, we then obtain the growth fraction of the tumor 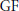, that is, the volume fraction that is proliferating, which varies in time and is determined from the tumor volume. When 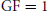 this represents a fully proliferating tumor. A key parameter is the volume 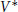 at which the tumor first experiences necrosis. The resulting model is similar to Eq. (1), but with only the growing fraction contributing to the tumor growth and with the drug affecting only living cells, thus



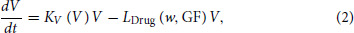





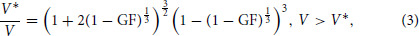



with 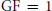 when 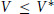. The growth equation, Eq. (2), is coupled to the equation for growing fraction 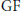, Eq. (3), and these two equations are solved together. In this model 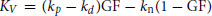 and the initial condition is 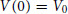. Specifically, 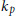 is the cell replication rate, 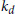 is the rate at which cells die in the proliferating region, and 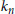 is the rate of breakdown and removal within the necrotic region. While the volume of the tumor 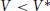, the entire tumor proliferates, and the model predicts exponential growth. As tumor size increases eventually 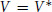 and the model switches to diffusion-limited growth, in which the tumor has a necrotic core surrounded by a proliferating growing rim, with the growth fraction of the tumor determined from Eq. (3; see Supplementary Information for further details).

The formulation Eqs. (2) and (3), which we term the diffusion-limited model, is of the same mathematical form as currently implemented. However, it is now possible to directly predict the growth fraction of the tumor. We will demonstrate the functionality of the description Eqs. (2) and (3) both for control datasets and for drug trial data. We focus here on preclinical trials of drug treatments. We take 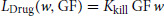, which is similar to the standard and broadly applicable exponential loss term (25); however, loss now only occurs in the proliferating compartment. We see that the mechanistic approach allows us to incorporate that cell cycle–specific pharmaceutical agents target proliferating cells. The pharmacokinetic description is in general compound specific. Here we consider CPT-11 (Irinotecan) which has been shown to be adequately described by an exponential model (14). We thus set for the fitting 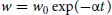, with 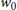 the known administered dose and 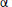 the decay rate, leaving 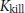 as the single parameter to be fitted describing drug effects. With the half-life of Irinotecan being 12 hours, 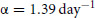.

### Numerical Implementation

Equations (2) and (3) form a growth law and two constraints which can be numerically solved as an algebraic differential equation. However, given current fitting protocols it is easier to work with a system of differential equations. This may be obtained from Equations (2) and (3) by differentiating the constraint Equation (3). All routines are implemented within MATLAB2019a (MathWorks) and are made available in GitHub at https://github.com/DrAdamNasim/Diffusion_Limited_Cancer_Growth_Model. Because of the significant intersubject heterogeneity, we use a nonlinear mixed effects fitting approach (NLME) to fit both the population and individual subject parameters using the stochastic approximation expectation-maximization algorithm (see Supplementary Data).

### Datasets Used for Model Validation

Control data are from AstraZeneca mouse-derived CDXs for two cell-lines SW620 (29 mice), epithelial colorectal adenocarcinoma, and Calu6 (178 mice), epithelial lung adenocarcinoma. Longitudinal time series of subcutaneous tumor volume were calculated by manual measurement of length and width using calipers (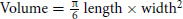). With regards treatment data, we consider SW620 CDXs (95 mice) which were treated with weekly doses of 50 mg/kg of CPT-11 (Irinotecan) either three times on days 1, 8, 15 (protocol 1, 68 mice), or four times during the experiment on days 1, 8, 15, 22 (protocol 2, 18 mice) or 4, 11, 18, 25 (protocol 3, 9 mice).

In addition, we use the Novartis PDX dataset, which is the largest publicly available database of PDX control data (26). The Novartis data contains 226 mice with six different tumor types: breast carcinoma 39 mice, non–small cell lung carcinoma 28 mice, gastric cancer 44 mice, colorectal cancer 45 mice, cutaneous melanoma 33 mice, and pancreatic ductal carcinoma with 37 mice.

All animal studies in the United Kingdom were conducted in accordance with the UK Home Office legislation, the Animal Scientific Procedures Act 1986, and with AstraZeneca Global Bioethics Policy.

### Data Availability

All codes are made freely available in GitHub at https://github.com/DrAdamNasim/Diffusion_Limited_Cancer_Growth_Model.

## Results

### Demonstration of the Functionality of the Model in Fitting Preclinical Data

**FIGURE 2:**
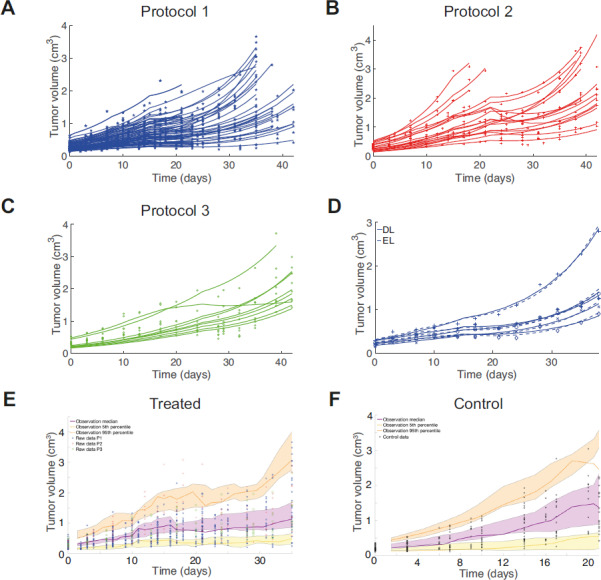
**A–C**, Fits of the diffusion-limited model to CDX treatment data for protocols 1, 2, and 3, respectively. Treatment protocols are defined in the Materials and Methods. **D**, Fit of both the diffusion-limited and exponential-linear models to five representative datasets from protocol 1. **E** and **F**, VPCs of both the treated and control CDX data, respectively. The VPC is based on 1,000 simulations, the shaded regions represent the 95% confidence intervals of the 5th (yellow), 50th (purple), and 95th (orange) percentiles of the simulated data. The experimental data median, 5th and 95th percentiles are marked (obtained using rolling average).

To demonstrate the fitting of the diffusion-limited model to longitudinal data, we consider the AstraZeneca CDX data in Fig. 2. In Fig. 2A–C, we show the individual fits of the diffusion-limited model to the experimental data separated by protocol. The diffusion-limited model is observed to fit well across the range of curves. Similarly, qualitatively good fits are observed for the other CDX and PDX control datasets (Supplementary Fig. S1). To further demonstrate the quality of fit, we perform a visual predictive check (VPC; ref. 27). This confirms that the diffusion-limited model captures the full range of dynamics (Fig. 2E and F). Similar results are also obtained from VPCs for the other control PDX and CDX datasets (Supplementary Fig. S2).

Looking quantitatively at the NLME results (Table 2), we see a small residual mean square error. We further compare the diffusion-limited fit to results obtained from fitting one of the most widely used models, the exponential-linear model (2, 14, 28), which has the same number of parameters as the diffusion-limited model. The parameter estimation for both the exponential-linear and diffusion-limited models is given in Table 2. Figure 2D shows five representative fits. We clearly see the similarity in the model behavior. This similarity in the quality of fit is confirmed by the closeness of the Akaike information criterion number (−217 and −221, for the diffusion-limited and exponential-linear, respectively) and root mean square error (0.146 and 0.142, respectively; Table 2). A similar result is achieved when considering the control datasets. These results are summarized in Supplementary Tables S1 to S8. Given the typically limited time course of preclinical data, it has been shown across numerous studies that no model may be optimal across all datasets (3, 6, 9). However, the results indicate that the diffusion-limited model provides at least a similar standard of fit for typical data.

**TABLE 2:** NLME results of 10 runs for the full AstraZeneca CDX dataset of 95 mice for the diffusion-limited (DL) and exponential-linear (EL) models

Model	Parameter	Definition	Value	iiv^a^	rmse	AIC
DL	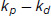	Net growth rate (day^−1^)	0.12	0.02	0.146	−217
	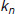	Necrotic loss rate (day^−1^)	0.05	0.03		
	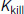	Drug potency (kg mg^−1^ day^−1^)	5.7 × 10^−3^	2.4 × 10^−4^		
	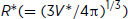	Critical radius (cm)	0.41	0.07		
	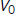	Initial volume (cm^3^)	0.25	0.11		
EL^b^	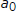	Exponential growth rate (day^−1^)	0.08	0.25	0.142	−221
	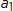	Linear growth rate (day^−1^)	0.31	0.06		
	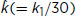	Transient death rate (day^−1^)	24	6.2		
	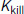	Drug potency (kg mg^−1^ day^−1^)	1.2 × 10^−3^	4 × 10^−4^		
	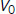	Initial volume (cm^3^)	0.29	0.13		

^a^Interindividual variability (iiv) quantified by the SD.

^b^In EL model, 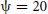 as fixed parameter.

### Diffusion-limited Model Predicts Growth Fraction Dynamics with Tumors

**FIGURE 3:**
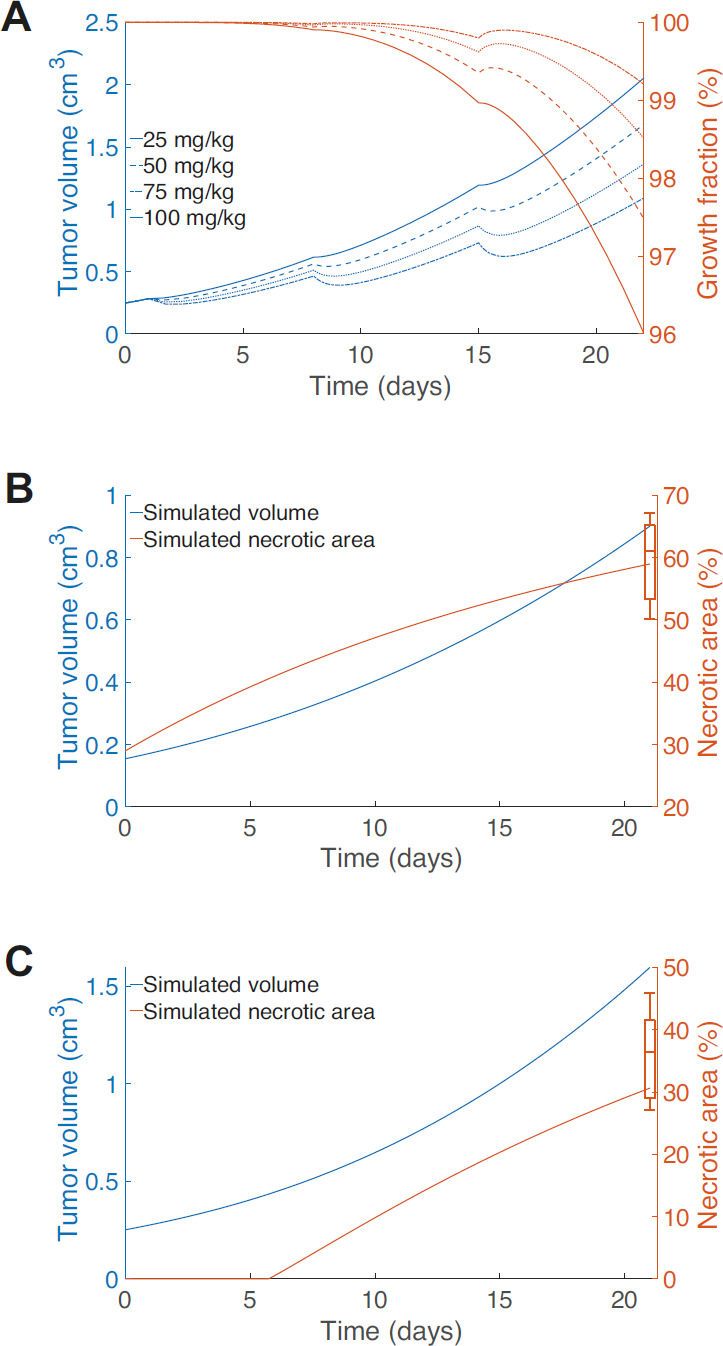
**A**, Simulated tumor response (blue) along with the predicted growth fraction (red) for dosing strengths 25, 50, 75, and 100 mg/kg (blue: top to bottom curves, respectively, red: bottom to top curves, respectively) dosed on days 1, 8, and 15. **B**, and **C**, Simulated tumor dynamics for the CDX xenografts [SW620 (**B**) and Calu6 (**C**) cell lines, simulated curves using population parameters from Supplementary Tables S1 and S2. The endpoint box plots are derived from histologic examination of necrotic area for eight SW620 xenografts and 10 Calu6 xenografts].

As discussed, the rate equation predicts the growth fraction 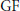 through Eq. (3). Thus, in addition to being able to fit the model to longitudinal measurements of tumor size, we can now predict how the growth fraction changes, including in response to treatment. We show, for example, in Fig. 3A that those tumors showing greatest response (dosing strengths 75 and 100 mg/kg) are predicted to have the largest growth fraction, with consequent implications for treatment. The growth fraction dynamically alters as the tumor volume changes, that is, as the tumor volume decreases so the diffusion equation predicts that more of the tumor will have access to nutrient responsively increasing the growing fraction accordingly.

The growth fraction of tumors is normally unavailable due to the difficulties of accessing this information *in vivo*. However, at the termination of xenograft experiments, it is possible to obtain growth fraction data through histology, although this is not routinely done. We consider independent datasets for the necrotic area for untreated SW620 and Calu6 cell lines obtained from histology of bisected tumors (Ki-67 staining). The total volumetric growth fraction 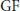, which varies in time, and this and the necrotic area of the cross-section are related by 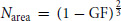. We simulate tumor dynamics for a tumor using the population parameters obtained from the mixed effects fitting of CDX data (Supplementary Tables S1 and S2). These simulations predict a necrotic area at the end of the experiment for both the SW620 cell line and the Calu6 cell lines that fits the experimental data (lying within the range of the data in Fig. 3B and C). In this case, the necrotic area data are an independent dataset. We could alternatively have used this data to further calibrate the model parameters. This highlights one of the benefits of a mechanistic model, that we can utilize other sources of information to better calibrate the model by way of multimodal fitting.

### Incorporation of Spatial Gradients in Drug Concentration

In fitting the CDX treatment data (Fig. 2), we assumed that the drug was available throughout the tumor at the same concentration, although of course the killing effect was only experienced by the proliferating cells. The drug concentration thus decreased in time but without spatial variation. For small molecule drugs, such as Irinotecan, this is likely a good approximation with good fits being obtained to the data. Indeed, nonspatial tumor kill models have been shown to provide fit to clinical treatment data well indicating broad applicability (25). However, for significantly larger molecule drugs, or for engineered drug delivery systems, the ability to deliver the drug to the tumor core by diffusion is likely to be restricted. Indeed, research into antibody–drug conjugates (ADC) already report this shielding effect as a potentially significant issue in treatment effectiveness (29).

The spatial distribution of drugs in the tumor can be incorporated in the model framework using the same diffusive framework as for nutrients. This extended model, while more complex, is equally straightforward to numerically implement requiring a single additional integration. As a specific example, we consider the case where cell loss only occurs in proliferating cells. In this case, the cell loss is integrated over the proliferating compartment so that



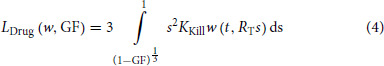



Where 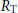 is the tumor radius, with for a spherical tumor 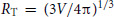. When the drug concentration 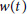 is the same throughout the tumor we recover 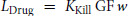 from Equation (4). For a drug diffusing into the tumor, however, the distribution is not constant and in this case the concentration in the proliferating region (where the drug acts) is (see Supplementary Data)



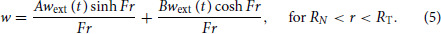



For 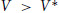, where 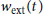 is the time varying applied drug concentration. The additional parameters 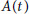 and 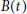 are known and defined in terms of 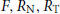 and 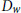, see Supplementary Information. This reduces to 
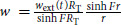
 when 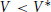 and 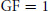. The parameter 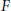 effectively quantifies the depth of penetration of the drug. A larger 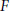 corresponds to more limited drug penetration, while as 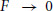 we regain a uniform drug distribution throughout the tumor. 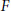 large corresponds to a surface kill. In terms of the other parameters 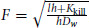, where 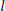 is the drug decay rate, 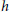 the drug efficiency, and 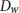 the drug diffusivity.

**FIGURE 4:**
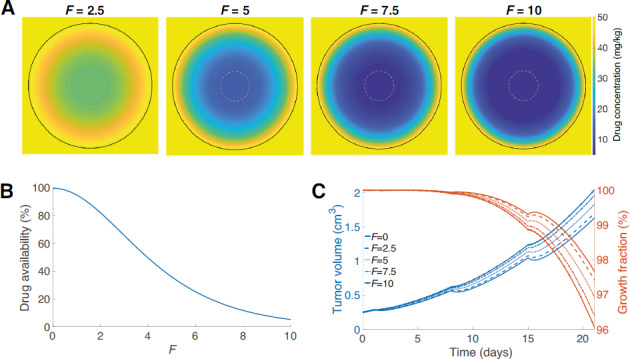
**A**, Heatmap of the simulated internal drug distribution within the tumor immediately following dosing on day 15. The grey dashed circle shows the predicted necrotic region. The tumor edge is indicated by the black solid line. **B**, Percentage of drug concentration reaching inner necrotic radius (corresponding to gray circle in **A**) as a function of drug localization parameter 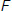. **C**, Tumor volume (blue) and growth fraction (red) dynamics for dosing strength fixed at 50 mg/kg dosed on days 1, 8, 15, for 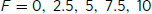 (blue: bottom to top curves, respectively, red: top to bottom curves, respectively). 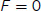 describes full drug penetration, with increasing 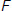 corresponding to a reduction in drug penetration. At 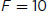, the drug effect is largely restricted to the surface of the tumor.

The solution 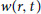 is then substituted into Eq. (4) to determine 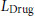. Although more complex than the previous model, it is easily implemented numerically. Heatmaps showing drug concentration within the tumor clearly demonstrate the effect of 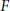 (Fig. 4A) As 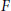 increases the amount of drug in the tumor drops significantly, indeed by the time 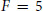 the concentration at the inner necrotic radius drops to 34% percent of its concentration at the outside edge (Fig. 4B). Exploring the effect of spatial drug distribution, we plot a representative solution of the spatial drug model (Fig. 4C). We see in the tumor growth curves (blue lines, Fig. 4C) that as 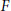 increases and the drug penetration is reduced (going from bottom to top) that the effect of the drug on the tumor growth is reduced, with less response at each dose. With greater drug penetration (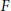 small), a greater effect is observed with faster dynamic rebounds. Overall, it is clear tumor response will vary depending on the drug distribution.

## Discussion and Conclusions

We present a mathematical model for tumor growth and treatment that is based on a mechanistic description of nutrient limited growth. Despite being derived from spatial partial differential equations, we show that these equations can be reduced to a growth law which is not significantly more complex than those currently used for preclinical drug trials. The work extends the growth modeling options available for preclinical drug trials enabling spatial effects to be explicitly taken into account.

The development of a mechanistic approach has several advantages over more phenomenological growth laws. Perhaps most significantly, in its current form the model allows for the dynamic prediction of the growing fraction of the tumor, accounting for cell loss from, for example, hypoxia. As most cytotoxic agents target only actively proliferating cells, tracking the growth fraction has potentially significant implications for treatment dynamics. This is only enhanced by the increasingly clear role hypoxia has in inducing downstream biological processes which directly promote tumor resistance to treatment (30–34). We have focused on preclinical trials of drug treatments and similar therapeutics. To model other types of cancer therapies, such as immunotherapies, the model would have to be adapted. However, the underpinning growth model could be incorporated into other modeling frameworks with different models of treatment.

By considering a range of different datasets including PDX and CDX data, we have shown that the diffusion-limited model fits the longitudinal volume data comparably with current industry-standard models. For independent datasets containing growth fraction data at termination, we also predict a growth fraction commensurate with that observed. This without presenting significant increased complexity in the numerical solution. In addition, the diffusion-limited model parameters are all based on observable phenomena: net proliferation rate, maximum volume before necrosis begins, initial volume and cell loss through necrosis. The introduction of spatial modeling into a growth law framework has also enabled us to present an additional extension to the model which enables the spatial distribution of drug throughout the tumor to be explicitly modeled. The indicative results obtained demonstrate the importance of accounting for this in preclinical studies where there is evidence of reduced penetration into the tumor. Indeed, the increasing importance of mAb treatments, with their reduced diffusivity, and ADCs (35) will only increase the importance of spatial modeling in preclinical trials.

## Supplementary Material

Supplementary Data Supplementary Calculations and Figures in one document

## References

[1] Yates JWT , Byrne H, Chapman SC, Chen T, Cucurull-Sanchez L, Delgado-Sanmartin J, . Opportunities for quantitative translational modeling in oncology. Clin Pharmacol Ther 2020;108:447–57. 32569424 10.1002/cpt.1963

[2] Simeoni M , Magni P, Cammia C, De Nicolao G, Croci V, Pesenti E, . Predictive pharmacokinetic-pharmacodynamic modeling of tumor growth kinetics in xenograft models after administration of anticancer agents. Cancer Res 2004;64:1094–101. 14871843 10.1158/0008-5472.can-03-2524

[3] Ribba B , Holford N, Magni P, Trocóniz I, Gueorguieva I, Girard P, . A review of mixed-effects models of tumor growth and effects of anticancer drug treatment used in population analysis. CPT Pharmacometrics Syst Pharmacol 2014;3:113. 10.1038/psp.2014.12 PMC4050233 24806032

[4] Murphy H , Jaafari H, Dobrovolny HM. Differences in predictions of ODE models of tumor growth: a cautionary example. BMC Cancer 2016;16:163. 26921070 10.1186/s12885-016-2164-x PMC4768423

[5] Kühleitner M , Brunner N, Nowak W-G, Renner-Martin K, Scheicher K. Best fitting tumor growth models of the von Bertalanffy-Puetter type. BMC Cancer 2019;19:683. 31299926 10.1186/s12885-019-5911-y PMC6624893

[6] Benzekry S , Lamont C, Beheshti A, Tracz A, Ebos JML, Hlatky L, . Classical mathematical models for description and prediction of experimental tumor growth. PLoS Comput Biol 2014;10:e1003800. 25167199 10.1371/journal.pcbi.1003800 PMC4148196

[7] Tjørve E , Tjørve KMC. A unifired approach to the Richards-model family for use in growth analyses: why we need only two model forms. J Theor Biol 2010;267:417–25. 20831877 10.1016/j.jtbi.2010.09.008

[8] Voulgarelis D , Bulusu KC, Yates JWT. Comparison of classical tumour growth models for patient derived and cell-line derived xenografts using the nonlinear mixed-effects framework. J Biol Dyn 2022;16:160–85. 35404766 10.1080/17513758.2022.2061615

[9] Marusic M , Bajzer Z, Vuk-Pavlovic S, Freyer JP. Tumor growth *in vivo* and as multicellular spheroids compared by mathematical models. Bull Math Biol 1994;56:617–31. 8054889 10.1007/BF02460714

[10] Ghaffari Laleh N , Loeffler CML, Grajek J, Staňková K, Pearson AT, Muti HS, . Classical mathematical models for prediction of response to chemotherapy and Immunotherapy. PLoS Comput Biol 2022;18:e1009822. 35120124 10.1371/journal.pcbi.1009822 PMC8903251

[11] Jing X , Yang F, Shao C, Wei K, Xie M, Shen H, . Role of hypoxia in cancer therapy by regulating the tumor microenvironment. Mol Cancer 2019;18:157. 31711497 10.1186/s12943-019-1089-9 PMC6844052

[12] Aleskandarany MA , Green AR, Rakha EA, Mohammed SE, Elsheikh SE, Powe DG, . Growth fraction as a predictor of response to chemotherapy in node-negative breast cancer. Int J Cancer 2010;126:1761–9. 19711345 10.1002/ijc.24860

[13] Gerlee P . The model muddle: in search of tumor growth laws. Cancer Res 2013;73:2407–11. 23393201 10.1158/0008-5472.CAN-12-4355

[14] Wilson S , Tod M, Ouerdani A, Emde A, Yarden Y, Adda Berkane A, . Modeling and predicting optimal treatment scheduling between the antiangiogenic drug sunitinib and irinotecan in preclinical settings. CPT Pharmacometrics Syst Pharmacol 2015;4:720–7. 26904386 10.1002/psp4.12045 PMC4759705

[15] d‘Esposito A , Sweeney PW, Ali M, Saleh M, Ramasawmy R, Roberts TA, . Computational fluid dynamics with imaging of cleared tissue and of *in vivo* perfusion predicts drug uptake and treatment responses in tumours. Nat Biomed Eng 2018;2:773–87. 31015649 10.1038/s41551-018-0306-y

[16] Sher A , Niederer SA, Mirams GR, Kirpichnikova A, Allen R, Pathmanathan P, . A quantitative systems pharmacology perspective on the importance of parameter identifiability. Bull Math Biol 2022;84:39. 35132487 10.1007/s11538-021-00982-5 PMC8821410

[17] Byrne HM , Chaplain MAJ. Growth of necrotic tumors in the presence and absence of inhibitors. Math Biosci 1996;135:187–216. 8768220 10.1016/0025-5564(96)00023-5

[18] Carrara L , Lavezzi SM, Borella E, DeNicolao G, Magni P, Poggesi I. Current mathematical models for cancer drug discovery. Expert Opin Drug Discov 2017;12:785–99. 28595492 10.1080/17460441.2017.1340271

[19] Mayneord WV . On the law of growth of Jensen's rat sarcoma. Am J Cancer Res 1932;16:841–6.

[20] Evans ND , Dimelow RJ, Yates JWT. Modelling of tumour growth and cytotoxic effect of docetaxel in xenografts. Comput Methods Programs Biomed 2014;114:e3–13. 23948442 10.1016/j.cmpb.2013.06.014

[21] Britton NF . Essential mathematical biology, Springer; 2003.

[22] Sutherland RM . Cell and environment interactions in tumour microregions: the multicell spheroid model. Science 1988;240:177–84. 2451290 10.1126/science.2451290

[23] Lowengrub JS , Frieboes HB, Jin F, Chuang Y-L, Li X, Macklin P, . Nonlinear modelling of cancer: bridging the gap between cells and tumours. Nonlinearity 2010;23:R1–R9. 20808719 10.1088/0951-7715/23/1/r01 PMC2929802

[24] Byrne HM . Dissecting cancer through mathematics: from the cell to the animal model. Nat Rev Cancer 2010;10:221–30. 20179714 10.1038/nrc2808

[25] Blagoev KB , Wilkerson J, Stein WD, Yang J, Bates SE, Fojo T. Therapies with diverse mechanisms of action kill cells by a similar exponential process in advance cancers. Cancer Res 2014;74:4653–62. 25183789 10.1158/0008-5472.CAN-14-0420 PMC8336537

[26] Gao H , Korn JM, Ferretti S, Monahan JE, Wang Y, Singh M, . High-throughput screening using patient derived tumor xenografts to predict clinical trial drug response. Nat Med 2015;21:1318–25. 26479923 10.1038/nm.3954

[27] Bergstrand M , Hooker AC, Wallin JE, Karlsson MO. Prediction-corrected visual predictive checks for diagnosing mixed effects models. AAPS J 2011;13:143–51. 21302010 10.1208/s12248-011-9255-z PMC3085712

[28] Terranova N , Magni P. TGI-Simulator: a visual tool to support the preclinical phase of the drug discovery process by assessing in silico the effect of an anticancer drug. Computat Methods Programs Biomed 2012;105:162–74. 10.1016/j.cmpb.2011.09.001 22005012

[29] Singh AP , Guo L, Verma A, Wong GG-L, Thurber GM, Shah DK. Antibody coadministration as a strategy to overcome binding-site barrier for ADCs: a quantitative investigation. AAPS J 2020;22:28. 31938899 10.1208/s12248-019-0387-x PMC8382310

[30] Riffle S , Hegde RS. Modeling tumor cell adaptations to hypoxia in multicellular tumor spheroids. J Exp Clin Cancer Res 2017;36:102. 28774341 10.1186/s13046-017-0570-9 PMC5543535

[31] Brown JM , Wilson WR. Exploiting tumour hypoxia in cancer treatment. Nat Rev Cancer 2004;4:437–47. 15170446 10.1038/nrc1367

[32] Strese S , Mårten M, Larsson R, Gullbo J. Effects of hypoxia on human cancer cell line chemosensitivity. BMC Cancer 2013;13:331. 23829203 10.1186/1471-2407-13-331 PMC3707755

[33] Zheng L , Kelly CJ, Colgan SP. Physiologic hypoxia and oxygen homeostasis in the healthy intestine. A review in the theme: cellular responses to hypoxia. Am J Physiol Cell Physiol 2015;309:C350–60. 26179603 10.1152/ajpcell.00191.2015 PMC4572369

[34] Däster S , Amatruda N, Calabrese D, Ivanek R, Turrini E, Droeser RA, . Induction of hypoxia and necrosis in multicellular tumor spheroids is associated with resistance to chemotherapy treatment. Oncotarget 2017;8:1725–36. 27965457 10.18632/oncotarget.13857 PMC5352092

[35] Goldmacher VS , Kovtun YV. Antibody-drug conjugates: using monoclonal antibodies for delivery of cytotoxic payloads to cancer cells. Ther Deliv 2011;2:397–416. 22834009 10.4155/tde.10.98

