# Supplementary material for: A Spatially Resolved Mechanistic Growth Law for Cancer Drug Development Predicting Tumor Growing Fractions
Source: Cancer Res Commun. 2022 Aug 2;2(8):754–61. doi: 10.1158/2767-9764.CRC-22-0032 (PMC10010375; doi:10.1158/2767-9764.CRC-22-0032)
Supplement: Supplementary Data — Supplementary Calculations and Figures in one document [file crc-22-0032-s01.pdf]

Supplementary Information:

A spatially resolved mechanistic growth law for cancer drug development predicting tumour growing fractions

A. Nasim, J. Yates, G. Derks and C. Dunlop

Tumour Growth Model Derivation

The paradigm diffusion-limited growth model of tumour growth [S1, S2, S3] is based on the solution of a diffusion model describing nutrient distribution within the tumour coupled to the equations of conservation of mass under the assumption of radial symmetry. We summarize the key points here.

The conservation of mass equation (with constant cell density) on the tumour domain is

$$\nabla \cdot \mathbf{u}(t, \mathbf{x}) = \Lambda(\mathbf{x}, t, w), \tag{S.1}$$

where $\mathbf{u}$ is the velocity field describing the motion of cells within the tumour and Λ is a growth function representing a mass source in proliferating regions and mass removal in necrotic regions. Cell proliferation increases the cellular mass which translates into tumour expansion and cell movement through the vector $\mathbf{u}$. For the drug treatment models below Λ depends on the drug concentration w as well.

In conjunction with the conservation of mass equation we solve the diffusion equation for the concentration of nutrient in the tumour which is denoted by c. As nutrient diffusion is a fast process compared with tissue growth, the quasi-steady approximation is valid leading to the diffusion equation in the form

$$D_c \nabla^2 c = k(c), \tag{S.2}$$

to be solved on the tumour domain, where $k(c)$ is the cellular uptake rate. Assuming a critical concentration $c = c_{\text{crit}}$ or equivalently a critical volume V^* above which necrosis is initiated due to lack of nutrient, the cellular uptake is constant when $V < V^*$ but above this size a necrotic region forms so that cell uptake only occurs for growing cells.

The parameter V^* can be expressed in terms of the other model parameters. To see this, consider the case where the tumour is small enough that all the cell proliferate and consume nutrient. Assuming radial symmetry, the diffusion equation (S.2) can be integrated twice to give the concentration of nutrient in the tumour as

$$c = c_0 - \frac{k_{\text{cons}}}{6D_c}(R_T^2 - r^2),$$

where k_{cons} is the assumed constant rate of consumption, R_T is the tumour radius, and c_0 is the concentration of nutrient outside the tumour. If necrosis occurs when $c = c_{\text{crit}}$, we see this will first occur at the centre of the tumour ($r = 0$), when the tumour radius is $R^* = \sqrt{6(c_0 - c_{\text{crit}})D_c/k_{\text{cons}}}$. Hence $V^* = 4\pi(R^*)^3/3$. The parameters D_c and k_{cons} enter the solution through V^* and only V^* is needed as a fit parameter of the model.

Consider the full model, after assuming radial symmetry, we introduce two dynamic radii: the overall tumour radius $R_T(t)$ and the radius at which the nutrient level drops low enough for necrosis to occur $R_N(t)$ (only relevant if R_T corresponds to a volume larger than V^*), see Fig. 1. The radius R_N can also be expressed in terms of the growth fraction:

$$GF = \frac{R_T^3 - R_N^3}{R_T^3}, \text{ hence } R_N = R_T(1 - GF)^{\frac{1}{3}}. \tag{S.3}$$

Under the assumption of spherical symmetry, the conservation of mass and

diffusion equations reduce to

$$\frac{1}{r^2}\frac{\partial(r^2 u_r)}{\partial r} = \begin{cases} -k_n, & \text{for } 0 < r < R_N, \\ k_p - k_d - K_{\text{kill}} w(t), & \text{for } R_N < r < R_T. \end{cases} \tag{S.4}$$

$$\frac{D_c}{r^2}\frac{\partial}{\partial r}\left(r^2 \frac{\partial c}{\partial r}\right) = \begin{cases} 0, & \text{for } 0 < r < R_N, \\ k_{\text{cons}}, & \text{for } R_N < r < R_T. \end{cases} \tag{S.5}$$

We can directly integrate the diffusion equation (S.5) on the domain $r \leq R_T$, with continuity conditions on c and $\partial c/\partial r$ across the inner boundary, boundedness at $r = 0$. Two final conditions come from that at $r = R_T$ the concentration is c_0 which is the known concentration of nutrient outside the tumour and that at R_N, $c = c_{\text{crit}}$ which is the minimal concentration before necrosis occurs (which as shown above may alternatively be expressed in terms of V^* the minimal volume). The solution for the concentration is then

$$c = \begin{cases} c_{\text{crit}}, & \text{for } 0 < r < R_N, \\ \frac{k_{\text{cons}}}{6D_c}\left(r^2 - \frac{2R_N^3}{r} + R_N^2\right) + c_{\text{crit}}, & \text{for } R_N < r < R_T. \end{cases}$$

Importantly, evaluating the solution for concentration at $r = R_T$ (where $c = c_0$) gives

$$\frac{6D_c(c_0 - c_{\text{crit}})}{k_{\text{cons}}} = R_T^2\left(1 + 2\frac{R_N}{R_T}\right)\left(1 - \frac{R_N}{R_T}\right)^2$$

which can be be rearranged using Equation (S.3) and the expression for V^* to give the growth fraction, which varies in time, as a function of the tumour volume through the equation

$$\frac{V^*}{V} = (1 + 2(1 - GF)^{\frac{1}{3}})^{\frac{3}{2}}(1 - (1 - GF)^{\frac{1}{3}})^3. \tag{S.6}$$

Finally, integrating the growth equation (S.4), in a similar manner gives a solution for the velocity $\mathbf{u}$. Evaluating this solution on $r = r_T$ where the velocity is $\frac{dR_T}{dt}(t)$ gives the growth law for the overall volume V, which after eliminating R_N using equation S.3 can be expressed in terms of the growth fraction as

$$\frac{dV}{dt} = K_V(V)V, \tag{S.7}$$

$$K_V = (k_p - k_d)GF - k_n(1 - GF). \tag{S.8}$$

Spatial drug distribution model

In a similar manner to the nutrient equation the spatial distribution of drug in the tumour is found by solving the diffusion equation for the drug concentration w on the tumour domain. We again assume a quasi-steady state due to drug diffusion being fast compared to tissue growth, a widely accepted assumption for cytotoxic chemotherapeutic agents [S4].

Assuming that drug uptake only occurs in the proliferative cells but with drug degradation continuing throughout the tumour, we get for the drug diffusion

$$D_w \nabla^2 w - lw = g(w, \mathbf{x}). \tag{S.9}$$

Here D_w is the diffusion constant of the particular compound in tumour tissue, l is the decay rate of the drug within the tumour. The function $g(w, \mathbf{x})$ is the form for the drug action which we assume here to be linear $g(w, \mathbf{x}) = \frac{K_{\text{kill}}}{h} w(t, \mathbf{x})$, within the proliferation part of the tumour, where K_{kill} is the drug kill, and $h > 0$ is the efficiency of the drug, this is a measure of how much drug is used in the killing process: $h \to 0$ indicates that large amounts of drug are required in the killing process whereas large h is the opposite. As for the nutrient equation, we obtain

$$\frac{D_w}{r^2} \frac{\partial}{\partial r} \left(r^2 \frac{\partial w}{\partial r} \right) = \begin{cases} lw, & \text{for } 0 < r < R_N, \\ lw + \frac{K_{\text{kill}}}{h} w, & \text{for } R_N < r < R_T. \end{cases} \tag{S.10}$$

We require that the drug concentration as well as its derivative are continuous throughout the tumour domain as well as that the concentration is finite at the tumour centre, and denote the external drug concentration at the tumour by $w(t, r = R_T) = w_{\text{ext}}(t)$, which is assumed known. This boundary value problem can be explicitly solved in terms of spherical harmonic functions [S5] which gives the distribution of drug concentration within the tumour when $V > V^*$ as

$$w(t, r) = \begin{cases} \frac{\bar{A} w_{\text{ext}}(t) \sinh Er}{Er}, & \text{for } 0 < r < R_N, \\ \frac{A w_{\text{ext}}(t) \sinh Fr}{Fr} + \frac{B w_{\text{ext}}(t) \cosh Fr}{Fr}, & \text{for } R_N < r < R_T. \end{cases} \tag{S.11}$$

where $E = \sqrt{\frac{l}{D_w}}$, $F = \sqrt{\frac{lh+K_{\text{kill}}}{hD_w}}$. The parameters $\bar{A}$, A and B are defined by

$$A = -MB = -\frac{MFR_T}{\cosh FR_T - M\sinh FR_T}, \bar{A} = \frac{E\left(A\sinh FR_N + B\cosh FR_N\right)}{F\sinh ER_N}$$

where $M = \frac{E\cosh FR_N\cosh ER_N - F\sinh FR_N\sinh ER_N}{E\cosh ER_N\sinh FR_N - F\cosh FR_N\sinh ER_N}$. Note that as the drug kill effect needs only be considered in the proliferative compartment we only require the solution for $w(r,t)$ only in $R_N < r < R_T$. When $V < V^*$ and $GF = 1$ the solution reduces to $w = \frac{w_{\text{ext}}(t)R_T\sinh Fr}{\sinh FR_T r}$.

Substituting for w from equation (S.11), proliferative compartment only, into the conservation of mass equation (S.4) and integrating as before gives the modified system

$$\frac{dV}{dt} = K_V V - L_{\text{Drug}}(w, GF)V, \tag{S.12}$$

$$K_V = (k_p - k_d)GF - k_n(1 - GF) \tag{S.13}$$

$$L_{\text{Drug}} = 3\int_{(1-GF)^{\frac{1}{3}}}^{1} s^2 K_{\text{kill}} w(t, R_T s)ds, \tag{S.14}$$

$$V^* = V(1 + 2(1 - GF)^{\frac{1}{3}})^{\frac{3}{2}}(1 - (1 - GF)^{\frac{1}{3}})^3. \tag{S.15}$$

Note that if $F = 0$ (full drug distribution, hence $w(t,r) = w_{\text{ext}}(t)$), the integral in (S.14) can be explicitly integrated and gives the relation for K_V as in (4).

Numerical implementation

The diffusion limited model Equations (2) to (3) (main paper) may be implemented as an algebraic differential equation (ADE), however we choose to work with it as a system of ODEs. To this end we take the time derivative of the nutrient constraint equation (3), this gives

$$\frac{dV}{dt} = K_V(V)V - L_{\text{Drug}}(w, GF)V, \tag{S.16}$$

$$K_V = (k_p - k_d)GF - k_n(1 - GF), \tag{S.17}$$

$$\frac{dGF}{dt} = -\frac{1}{3}(K_V - L_{\text{Drug}})\Big((1 - GF)^{1/3} + (1 - GF)^{2/3} - 2(1 - GF)\Big). \tag{S.18}$$

We solve (S.16)–(S.18) using MATLAB(2019a) using the ODE45 solver for the exponential phase and the ODE15S solver for the non-exponential phase using

an event solver to manage the switch between phases. A stiff solver is required due to the high stiffness that may occur during integration from high dose strengths.

The mixed effects data fitting was implemented within MATLAB(2019a) using the NLMEFITSA optimisation routine. (With the necrotic radius R^* used as a fit parameter rather than V^*.) This performs Monte Carlo simulation designed to converge to the maximum likelihood estimates of the parameters. Multiple initial parameter estimates were tested with no significant differences in either the parameter estimation, curve fitting or run times being observed. The initial parameter values used are listed in the GitHub code [S6]. A linear approach to the approximation of the log likelihood was chosen. This was due to it being much faster to converge to a solution compared to other approximate techniques (Gaussian quadrature and Importance sampling were also tested, but they did not give significantly different results and took far longer to run). The default Matlab constant error model is used. We used a probit transform for the parameter transform which is a natural way to bound the parameters (a logit transform could also have been used). In particular, this is useful to ensure large values for the kill parameter are not tested, which occurs when using the common log transform. Choosing large, unphysical values of the kill term result in increased stiffness of the system and longer run times. The probit transform is defined as

$$\text{Probit}(\phi) = \sqrt{2}\,\text{erf}^{-1}(2\phi - 1), \qquad \phi \in \Re,$$

where erf is the error function. This probit transform is appropriate for all parameters except the rate of damage parameter k_1 for the exponential-linear model. This parameter is required to be greater than one and so it was scaled as indicated in Table 2. Where standard deviations are calculated these are obtained from the individual parameters for each individual fit. These were transformed from the probit scale into the physical domain using the inverse probit transform and the standard deviation subsequently calculated.

References

[S1] J. S. Lowengrub, H. B. Frieboes, F. Jin, Y. L. Chuang, X. Li, P. Macklin, S. M. Wise, and V. Cristini. Nonlinear modelling of cancer: bridging the gap between cells and tumours. *Nonlinearity*, 23(1):R1–R9, 2010.

[S2] H. M. Byrne and M. A. Chaplin. Growth of necrotic tumors in the presence and absence of inhibitors. *Math. Biosci.*, 135(2):187–216, 1996.

[S3] N.F. Britton. *Essential Mathematical Biology*. Springer, 2005.

[S4] V. A. Levin, C. S. Patlak, and H. D. Landahl. Heuristic modeling of drug delivery to malignant brain tumors. *J. Appl. Biopharm.*, 8(3):257–296, 1980.

[S5] A. Abramowitz and I.A. Stegun. *Pocketbook of Mathematical Functions*. Harri Deutsch Verlag, 1984.

[S6] Adam Nasim. GitHub. `https://github.com/DrAdamNasim/Diffusion_Limited_Cancer_Growth_Model`.

[S7] H. Gao, J. M. Korn, S. Ferretti, J. E. Monahan, Y. Wang, M. Singh, C. Zhang, C. Schnell, G. Yang, Y. Zhang, O. A. Balbin, S. Barbe, H. Cai, F. Casey, S. Chatterjee, D. Y. Chiang, S. Chuai, S. M. Cogan, S. D. Collins, E. Dammassa, N. Ebel, M. Embry, J. Green, A. Kauffmann, C. Kowal, R. J. Leary, J. Lehar, Y. Liang, A. Loo, E. Lorenzana, E. Robert McDonald, M. E. McLaughlin, J. Merkin, R. Meyer, T. L. Naylor, M. Patawaran, A. Reddy, C. Röelli, D. A. Ruddy, F. Salangsang, F. Santacroce, A. P. Singh, Y. Tang, W. Tinetto, S. Tobler, R. Velazquez, K. Venkatesan, F. Von Arx, H. Q. Wang, Z. Wang, M. Wiesmann, D. Wyss, F. Xu, H. Bitter, P. Atadja, E. Lees, F. Hofmann, E. Li, N. Keen, R. Cozens, M. R. Jensen, N. K. Pryer, J. A. Williams, and W. R. Sellers. High-throughput screening using patient-derived tumor xenografts to predict clinical trial drug response. *Nat. Med.*, 21(11):1318–1325, 2015.

Model	Parameter	Definition	Value	iiv	rmse	AIC
DL	$k_p - k_d$	Net growth rate(day^{-1})	0.131	0.021	0.086	-150
	k_n	Necrotic loss rate (day^{-1})	0.021	0.012		
	$R^*(= (3V^*/4\pi)^{1/3})$	Critical Radius (cm)	0.251	0.007		
	V_0	Initial Volume (cm^3)	0.185	0.088		
EL	a_0	Exponential growth rate(day^{-1})	0.103	0.062	0.090	-139
	a_1	Linear growth rate(day^{-1})	0.157	0.327		
	V_0	Initial Volume(cm^3)	0.182	0.321		

Table S.1: NLME results of 10 runs for untreated SW620 CDX dataset 29 mice.

Model	Parameter	Definition	Value	iiv	rmse	AIC
DL	$k_p - k_d$	Net growth rate(day^{-1})	0.091	0.018	0.067	-1193
	k_n	Necrotic loss rate (day^{-1})	0.038	0.020		
	$R^*(= (3V^*/4\pi)^{1/3})$	Critical Radius (cm)	0.485	0.118		
	V_0	Initial Volume (cm^3)	0.255	0.111		
EL	a_0	Exponential growth rate(day^{-1})	0.086	0.025	0.072	-1197
	a_1	Linear growth rate(day^{-1})	0.203	0.058		
	V_0	Initial Volume(cm^3)	0.266	0.112		

Table S.2: NLME results of 10 runs for untreated Calu6 CDX dataset 178 mice.

Model	Parameter	Definition	Value	iiv	rmse	AIC
DL	$k_p - k_d$	Net growth rate(day^{-1})	0.054	0.190	0.056	-889
	k_n	Necrotic loss rate(day^{-1})	0.055	0.041		
	$R^*(= (3V^*/4\pi)^{1/3})$	Critical Radius (cm)	0.458	0.086		
	V_0	Initial Volume (cm^3)	0.259	0.035		
EL	a_0	Exponential growth rate(day^{-1})	0.062	0.019	0.057	-922
	a_1	Linear growth rate(day^{-1})	0.040	0.025		
	V_0	Initial Volume(cm^3)	0.264	0.031		

Table S.3: NLME results of 10 runs for untreated PDAC PDX dataset 37 mice.

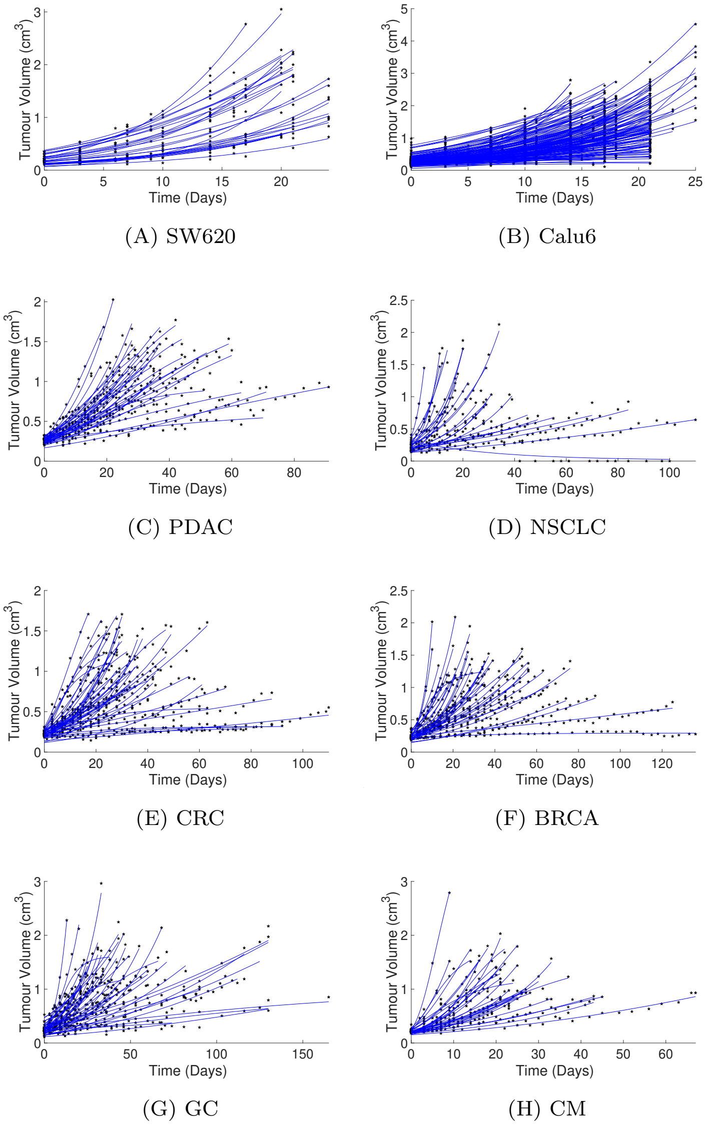

(A) SW620 (B) Calu6

(C) PDAC (D) NSCLC

(E) CRC (F) BRCA

(G) GC (H) CM

Figure S.1: (a) to (h) show the fitted curves generated via the NLME optimisation for the diffusion limited model for all untreated data sets considered. (A) and (B) CDX control data sets from AstraZeneca for SW620 and Calu6 cell lines. (C)–(H) PDX control data sets from Novartis dataset [S7]. The stars indicate the volume data and the solid lines the simulated tumour growth curves.

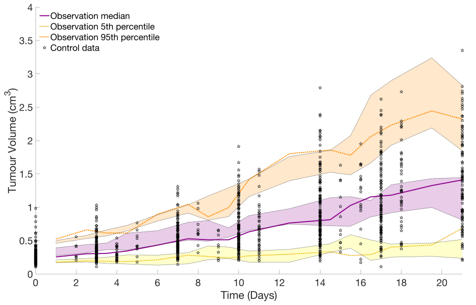

(A) VPC CDX Calu6

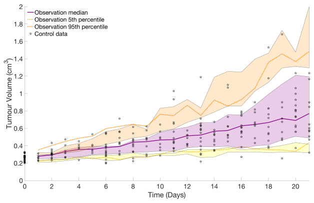

(B) VPC PDX PDAC

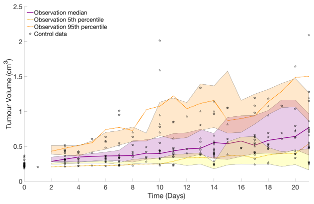

(C) VPC PDX BRCA

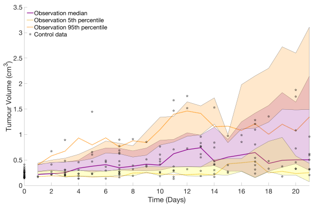

(D) VPC PDX NSCLC

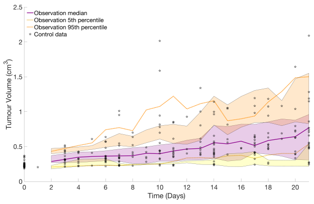

(E) VPC PDX GC

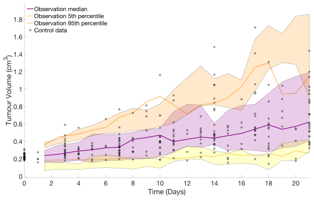

(F) VPC PDX CRC

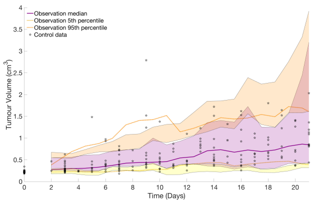

(G) VPC PDX CM

Figure S.2: (A)–(G) shows the visual predictive check (VPC) for the control experiments considered in Fig. S.1. The VPC for the SW620 CDX data is found in the main paper. In all figures the orange, purple and yellow clouds indicate the 95th, 50th and 5th percentile confidence intervals respectively.

Model	Parameter	Definition	Value	iiv	rmse	AIC
DL	$k_p - k_d$	Net growth rate(day^{-1})	0.061	0.190	0.046	-1096
	k_n	Necrotic loss rate(day^{-1})	0.004	4×10^{-4}		
	$R^*(= (3V^*/4\pi)^{1/3})$	Critical Radius (cm)	0.6	0.086		
	V_0	Initial Volume (cm^3)	0.247	0.035		
EL	a_0	Exponential growth rate(day^{-1})	0.053	0.562	0.049	-1075
	a_1	Linear growth rate(day^{-1})	0.183	3.153		
	V_0	Initial Volume(cm^3)	0.246	0.054		

Table S.4: NLME results of 10 runs for untreated BRCA PDX dataset 39 mice.

Model	Parameter	Definition	Value	iiv	rmse	AIC
DL	$k_p - k_d$	Net growth rate(day^{-1})	0.085	0.052	0.068	-397
	k_n	Necrotic loss rate(day^{-1})	0.010	0.004		
	$R^*(= (3V^*/4\pi)^{1/3})$	Critical Radius(cm)	0.385	0.19		
	V_0	Initial Volume(cm^3)	0.218	0.068		
EL	a_0	Exponential growth rate(day^{-1})	0.062	0.067	0.076	-356
	a_1	Linear growth rate(day^{-1})	0.193	0.037		
	V_0	Initial Volume(cm^3)	0.220	0.061		

Table S.5: NLME results of 10 runs for untreated NSCLC PDX dataset 28 mice.

Model	Parameter	Definition	Value	iiv	rmse	AIC
DL	$k_p - k_d$	Net growth rate(day^{-1})	0.060	0.028	0.081	-513
	k_n	Necrotic loss rate (day^{-1})	0.002	0.006		
	$R^*(= (3V^*/4\pi)^{1/3})$	Critical Radius(cm)	0.413	0.140		
	V_0	Initial Volume(cm^3)	0.252	0.082		
EL	a_0	Exponential growth rate(day^{-1})	0.052	0.031	0.087	-500
	a_1	Linear growth rate(day^{-1})	0.057	0.049		
	V_0	Initial Volume(cm^3)	0.254	0.073		

Table S.6: NLME results of 10 runs for untreated GC PDX dataset 44 mice.

Model	Parameter	Definition	Value	iiv	rmse	AIC
DL	$k_p - k_d$	Net growth rate(day^{-1})	0.054	0.040	0.043	-1231
	k_n	Necrotic loss rate(day^{-1})	0.048	0.043		
	$R^*(=(3V^*/4\pi)^{1/3})$	Critical Radius(cm)	0.434	0.128		
	V_0	Initial Volume(cm^3)	0.223	0.173		
EL	a_0	Exponential growth rate(day^{-1})	0.061	0.034	0.047	-1196
	a_1	Linear growth rate(day^{-1})	0.045	0.034		
	V_0	Initial Volume(cm^3)	0.228	0.051		

Table S.7: NLME results of 10 runs for untreated CRC PDX dataset 45 mice.

Model	Parameter	Definition	Value	iiv	rmse	AIC
DL	$k_p - k_d$	Net growth rate(day^{-1})	0.048	0.051	0.050	-441
	k_n	Necrotic loss rate(day^{-1})	0.05	0.02		
	$R^*(=(3V^*/4\pi)^{1/3})$	Critical Radius(cm)	0.53	0.064		
	V_0	Initial Volume(cm^3)	0.30	0.034		
EL	a_0	Exponential growth rate(day^{-1})	0.105	0.080	0.051	-467
	a_1	Linear growth rate(day^{-1})	0.086	0.057		
	V_0	Initial Volume(cm^3)	0.214	0.030		

Table S.8: NLME results of 10 runs for untreated CM PDX dataset 33 mice.
